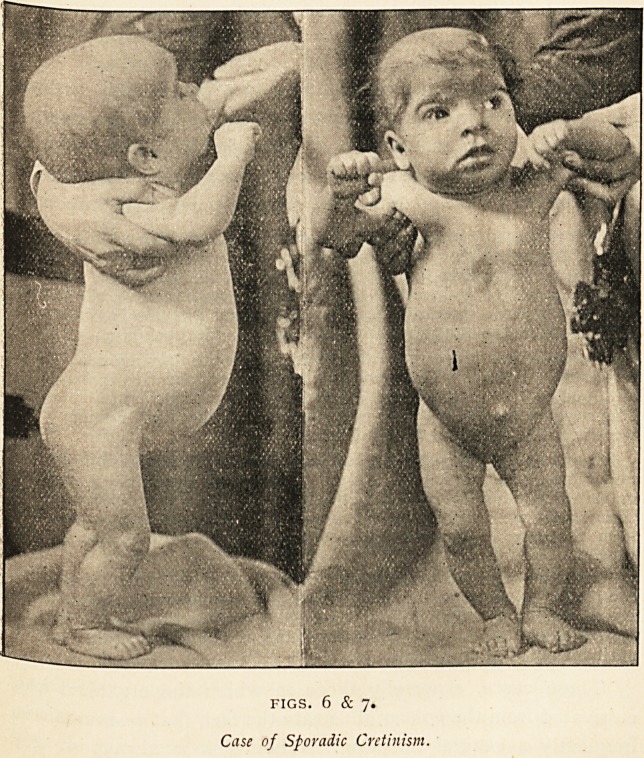# Achondroplasia
1A Paper read before the Bath and Bristol Branch of the British Medical Association, November 30th, 1898.


**Published:** 1899-03

**Authors:** Charles E. S. Flemming


### ACHONDROPLASIA.[1]

BY

Charles E. S. Flemming, M.R.C.S., L.R.C.P.

Achondroplasia, more pleasantly but less properly called fœtal rickets, though not common, is of sufficient importance in its after-effects to be of more interest than a mere pathological curiosity. It is a disease of intra-uterine life, characterised by great shortness of the limbs, which are bent and markedly out of proportion to a fully developed trunk, and an enlarged abdomen. The head is rather large, and there is considerable thickening of the skin of the whole body.

Mrs. H., an anæmic, but otherwise healthy woman, mother of one healthy child, was in the eighth month of her pregnancy confined of a stillborn male child; the second stage was prolonged, and owing to the soft state of the head there was some

[1] A Paper read before the Bath and Bristol Branch of the British Medical Association, November 30th, 1898.

difficulty in diagnosing the presentation, which was first vertex. The child died, apparently during labour. The placenta was normal in appearance. The child had a remarkable appearance. (Fig. 1.) Except for the enlarged belly, there was little to note in the trunk; the head was rather large, but so soft and shapeless that the nurse's description of it as "real pudden-headed" was not hyperbolical; its condition is well shown by the mark of the string which had to be used to hold it in position for photographing. Only towards the base of the skull could any resisting bone be felt. The face was expressionless and heavy, the nose wide and flat, and the skin about the face and neck appeared very thick, so much so that in the neck it was in a large fold. The most marked deformity was in the limbs; these were very short in proportion to the trunk, markedly curved and bent, with relatively small hands and feet. The width from shoulder to shoulder was normal, but the arms were very short and deformed; the arm and forearm both bent to nearly a right angle, the concavity being forwards and inwards; in addition the wrists were strongly pronated. For the purpose of photographing, the body was "displayed proper," as they say in heraldry, so that the pronation is less marked in the picture than it was in reality. The hands were very small, but not deformed.

FIG. 1.

*Photograph of Stillborn Child affected with Achondroplasia. Reduced one-fourth.*

The legs, like the arms, were short and bent; the curve of the thigh was a double one, and not so acute as in the arm; the most marked curve was at about the junction of the lower and middle thirds, and had its concavity forwards; above this was a slight curve backwards, and a little inwards. The legs had the most marked deformity of all, being bent backwards to less than a right angle. The feet were in a position of equino-varus, due to deformity rather of the leg than of the tarsus.

The skin was everywhere thickened, and on section it was clear that this thickening was due to an increase in the deeper areolar layer, and not merely to an increase in the subcutaneous fat; and in the penis, where there is no fat, the skin was markedly thick. The tongue appeared too large for the mouth, as it does in those cases of sporadic cretinism where there is a great increase of connective tissue at its root.

FIG. 2.

*Photograph of longitudinal section of Femur and Tibia from the same case. Four-fifths actual size.*

There was no gross nor microscopical change in the thyroid, and all the organs of the thorax and abdomen appeared normal. The cartilaginous epiphyses of the long bones (Fig. 2) were enlarged in every direction, and did not present the usual white line at their junction with the diaphyses.

The bones were easily cut with a knife from end to end; the shafts of the long bones had merely a thin casing of bone (owing to the obliquity of the section, this appears thicker in the femur than it really was), and the cancellous bone inside felt only gritty to the knife. The cranial bones were no stiffer than a sheet of writing paper. The clavicle and ribs, though soft, were normal in shape and size, and the pelvis presented no marked deformity.

When examining the minute anatomy of the cartilage in a section of a normal epiphysis at the same period of fœtal life, we see from above downwards the oval masses of proliferating cartilage cells, the calcification of their capsules and the formation of the primary areolæ, then the excavation of the secondary areolæ in this calcified cartilage by the osteoclasts, and finally the deposition of new bone on the walls of the secondary areolæ by the osteoblasts. Now in a section of the same bone, tibia, from the case of achondroplasia, we see a sparseness of the proliferating cartilage cells, and only a few calcified capsules with shrunken cells, *i.e.*, a deficiency of the primary areolæ, and the proliferating cells abut immediately on a mass of osteoblasts and osteoclasts in which are but few calcified or ossifying trabeculæ; it is noteworthy that the process, such as it is, is fairly regular. Further on, in the shaft, we find great quantities of these round cells and a few bony trabeculæ, but the periosteal layer of bone appears fairly normal.

In the section of a parietal bone we see greatly delayed ossification. The appearance of the cartilage suggests no loss of power to grow, but an almost complete incapacity to carry out its bone-forming duties; the cells grow, but refuse to calcify, and the round cells crowd round and absorb them, the deficient calcification apparently interfering with the ossifying function of the osteoblasts.

It would seem then, that while owing to some dystrophy the skeleton maintained for a long while its embryonic form, there was some special affection of the epiphyseal cartilage whereby its ossification was not only delayed but reduced almost to a minimum.

While then the periosteum is forming bone, the cartilage on which the length of the bone depends is inactive, the consequence being that although the bone has a normal, or nearly normal, transverse diameter, it is at the same time greatly reduced in length. Bones in the growth of which the cartilage takes but a small part, such as the clavicle and the ribs, are not shortened, and bones developed in membrane are of natural size.

The lesions of the long bones produced by the delay in ossification are so symmetrical and of such a nature that they cannot be considered as due to any external accidental cause. They are evidently due entirely to muscular action. (Fig. 3.) The biceps has bent forwards the humerus, and the flexors and pronators of the wrist have rotated and flexed the radius and ulna.

FIG. 3.

*Radiogram of the same case. Reduced one-fourth.*

In the lower extremity, as a longitudinal section shows, there is a forward curve of the femur due undoubtedly to the action of the quadriceps extensor; the slight curve above, backwards and inwards, is, I suppose, produced by the adductors of the thigh and flexors of the leg. The tibia is evidently doubled up by the gastrocnemius and soleus.

The following short description of this disease is gathered from published papers, the most important being one by Dr. Porak[1] of Paris. Achondroplasia is characterised by lesions of the skeleton which are symmetrical and chiefly in the long bones; these are thick, short, hard, and compact, and where bent the bending is always in the diaphyses and in the same direction. It is not a question of local, or rather special lesions of the bones, for these are accompanied by profound nutritional lesions, in particular a great thickening of the skin.

The affection does not show itself in the trunk or the head, except at the base of the skull, which is contracted, and has the bones prematurely united. Some cases are, however, hydrocephalic. It is a disease which comes on and completes its evolu-

[1] *De l'Achondroplasie.*

tion in the earlier months of pregnancy, so that when the child is born the lesions are cured and the initial disorders escape observation. However, one authority says that the disease may be active until the twenty-sixth week.

The bone when formed is very hard, and the epiphyses are greatly enlarged. The whole appearance of a case of achondroplasia may be best described as cretinoid, and the disease is probably closely allied to, if not a phase of, sporadic cretinism. The chief, if not the only, difference between my case and those already described is the length of time during which the disease remained active. When it has ceased early, we should expect shortening, but little bending, of the bones; but when it has lasted long and the muscles grown strong, the bending of the bones will probably be increased in proportion to the lateness of recovery.

It is necessary to distinguish these cases from inherited syphilis and from rickets. In the former there is a great overgrowth at the diaphyso-epiphyseal junction, where later the tissues degenerate, and we may then find dislocation of the epiphyses. There are characteristic osteophytic productions under the periosteum, and the long bones far from being shortened may actually be lengthened. There are generally other concomitant signs of syphilis. Rickets as a rule does not occur until about the second year of life. Kassowitz, however, says that it is a frequent occurrence in stillborn or early dying children, but passes unnoticed, as it is not perceived until a child begins to walk, but it does not seem to be obvious why the lesions of the ribs and arms should not be observed.

In rickets the lesions are not so symmetrical, the ribs are nearly always affected; the curves in the long bones may be merely exaggerations of the natural curves, and most often are due to pressure, and they are accompanied by actual, not merely relative, thickening of the bone; as a rule the curves that occur early are near the epiphyses, and the lesions are often isolated. The microscopic appearance of a rickety epiphysis is characteristic; there is an increased proliferation of cartilage cells, the line of junction with the diaphysis is markedly irregular, the secondary areolæ are large, vascular, and irregularly disposed, islets of cartilage are seen in the osteoid trabeculæ and in the medullary cavities, and medullary cavities occur in the epiphyseal cartilage.

---

In the discussion which followed, Dr. F. H. EDGEWORTH said: Achondroplasia is probably not such a very rare disease, though cases do not come under observation for treatment. I know by sight at least four persons in Bristol who are affected with it, in addition to the one whose photographs are here reproduced (Figs. 4 and 5), and who was admitted to the Bristol Royal Infirmary eighteen months ago for

FIGS. 4 & 5

*Case of Achondroplasia.*

gastro-intestinal catarrh by the house physician, Dr. Stack, who recognised the skeletal condition.

The patient, now 59 years of age, is one of a numerous family, the other members of which are of normal stature. He has no children. His height is 4 feet 8 inches, and the pictures show that this shortness of stature is due to deficiency in the length of the long bones of the legs. The long bones of the arms are similarly very short. The trunk shows no abnormality. The muscles are very well developed, through having short bellies and tendons. Skiagrams of the limbs show that the bones are of normal transverse diameters, and the only defects observable are a shortness of the diaphyses and a slight exaggeration of the ordinary curves. The shape of the head is peculiar. There is a short basicranial axis associated with a globular vault, great vertical height and prominent forehead, producing, as it has been said, a pouter-pigeon aspect. (This conformation of head has been shown by Thomson and Symington[1] to be a direct result of the early synostosis of the basicranial axis, and the consequent expansion of the growing brain in other directions.) There are no myxœdematous features: the skin is not thickened and is of healthy tint, the thyroid is of normal size, the tongue is normal, there are no supra-clavicular masses of fat, the abdomen is normal, and there is not the least trace of mental hebetude—in fact the man is particularly quick in mental action and bodily movement.

The etiology of achondroplasia is obscure. It was shown by Thomson and Symington that the essential skeletal abnormalities of the disease are of two kinds—premature synostosis of the basicranial axis, and defective endochondral ossification, so that the diaphyses of long bones are shorter than usual.

It was also pointed out by these observers that the only condition to which achondroplasia has any resemblance is sporadic cretinism, in which precisely the same osseous abnormalities are present.

Figs. 6 and 7 of a case of sporadic cretinism, which was under my care a year or two ago, clearly show the similar changes in the shape of the head and in the length of the limbs.

Achondroplasia differs however from sporadic cretinism in that the thyroid body is normal, and in the majority of cases there is an absence of cretinoid features, *e.g.* enlarged tongue, supra-clavicular masses of fat, enlarged abdomen, myxœdematous condition of the skin, defective intellect. Mr. Flemming's case is exceptional in that these cretinoid features were present; indeed, were it not that the thyroid body was normal, one would be fully justified in calling it one of sporadic cretinism.

These facts lead to the following suggestion. It is well known that the thyroid body is originally a gland pouring a secretion into the alimentary canal. This function ceases early in the vertebrate family history and in the individual life-history of the higher vertebrates; and the only trace in adult life of this part played in the past is the foramen cæcum at the base of the tongue and an atrophied duct passing up in front of the hyoid bone, portions of which, persisting, may form troublesome mucus-secreting cysts. But another function is superimposed on the gland, by reason of which it continues in existence and develops—that of producing substances which, passing into the lymphatics and thence into the blood, keep the body in good health, and the failure of which from disease brings about a myxœdematous condition.

Now, in the usual cases of achondroplasia, *e.g.* the man above described, there can be but little doubt that the thyroid body ultimately performs its function of producing an internal secretion. The skeletal changes, however, indicate that probably during early fœtal life this function was absent or deficient. Achondroplasia, then, is possibly due to a retardation in the development of this function. The cases may thus be cretins in early intra-uterine life; and whilst, on the development of the secondary function of the thyroid, the tissue

[1] *Rep. Lab. Roy. Coll. Phys., Edinb.*, 1892, iv. 237.

changes other than the osseous rapidly improve, the latter from their very nature persist and leave indelible traces of the events of the past.

Mr. Flemming suggests that in his case the disease had remained active much longer than usual. This suggestion, it may be remarked, is in harmony with the cretinoid condition of the child, and probably the "internal" secretion of the thyroid had not yet begun to be formed.

FIGS. 6 & 7.

*Case of Sporadic Cretinism.*

## Figures and Tables

**FIG. 1. f1:**
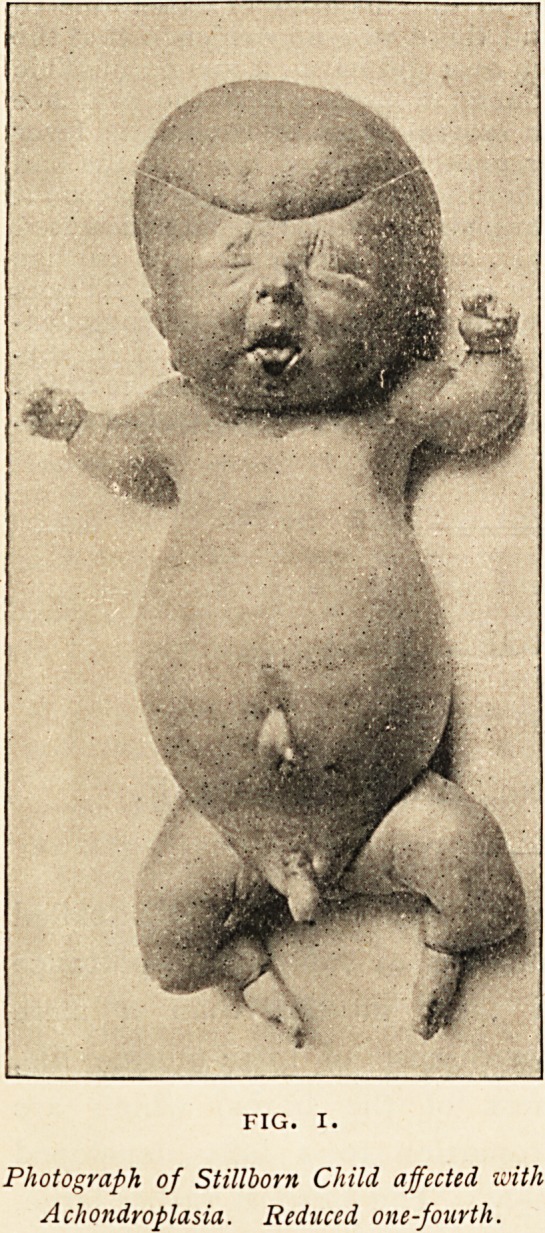


**FIG. 2. f2:**
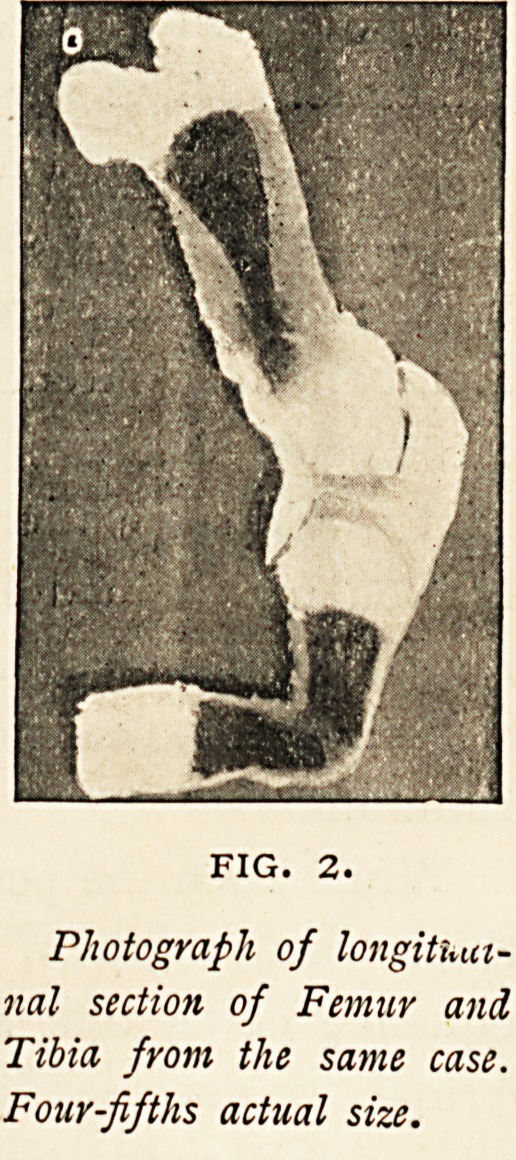


**FIG. 3. f3:**
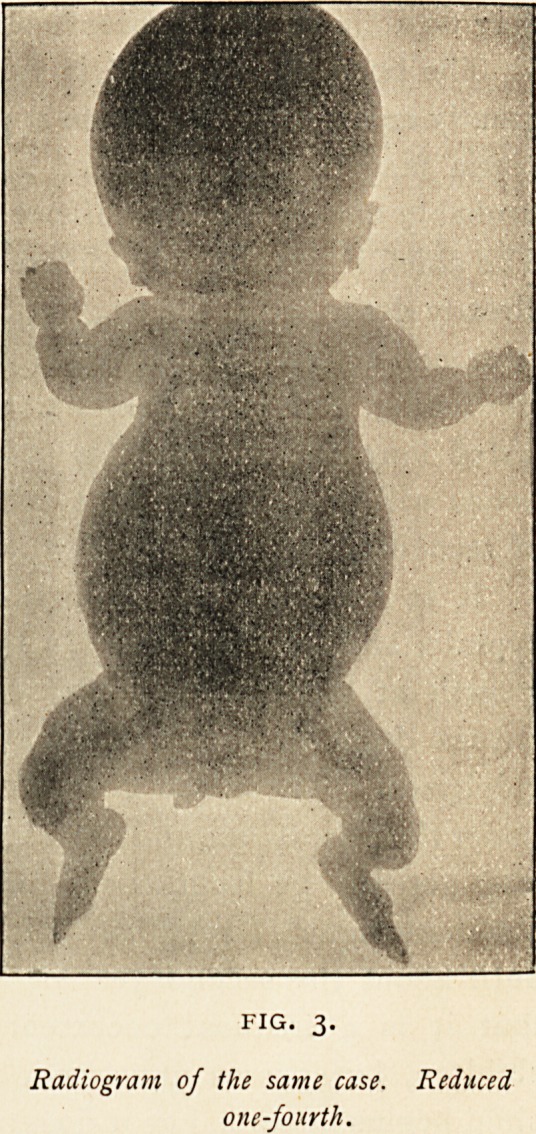


**FIGS. 4 & 5 f4:**
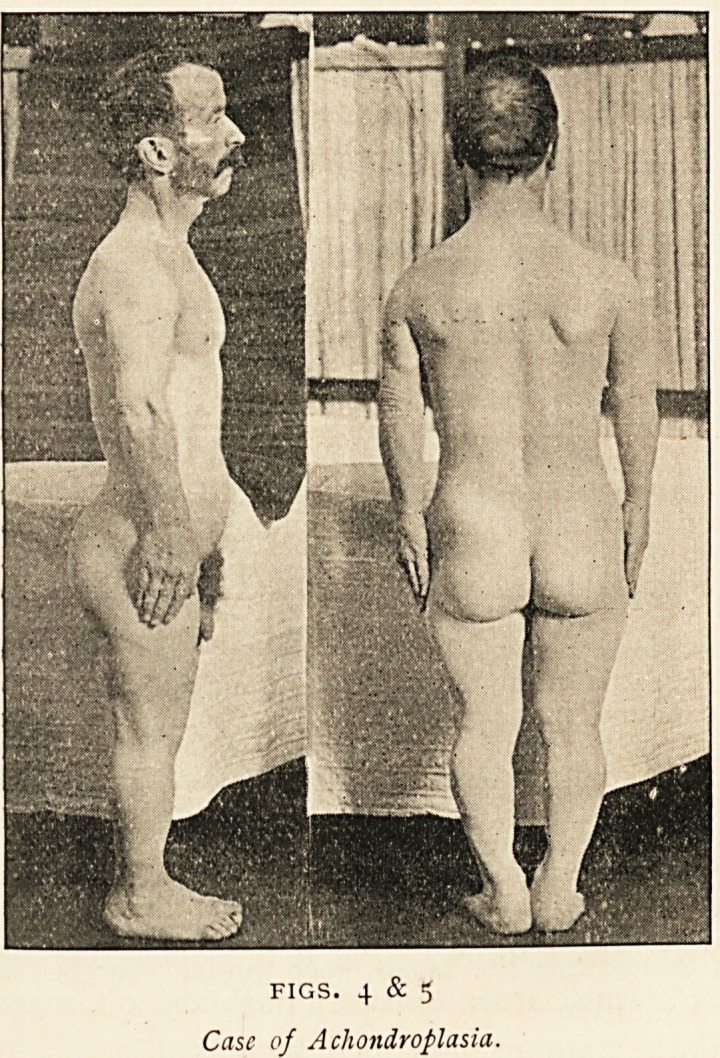


**FIGS. 6 & 7. f5:**